# Recurrent Oral Ulcers: Are They Horses or Zebras?

**DOI:** 10.7759/cureus.61908

**Published:** 2024-06-07

**Authors:** Vandana Bandari, Sai Rakshith Gaddameedi, Malay Rathod, Phani Bhavana Cherukuri, Jinsong Zhang, Shazia M Shah

**Affiliations:** 1 Internal Medicine, Bayhealth Medical Center, Dover, USA; 2 Internal Medicine, Rutgers Health-Monmouth Medical Center, Long Branch, USA; 3 Rheumatology, Bayhealth Medical Center, Dover, USA

**Keywords:** small vessel vasculitis, autoimmune, steroids, colchicine, genital ulcers, recurrent oral ulcers, oral ulcers, behcet disease

## Abstract

Oral ulcers are one of the most common complaints seen by general practitioners in their offices. Recurrent aphthous stomatitis affects roughly 20% of the general population. When ulcers persist despite conventional treatment, it is crucial to consider systemic diseases such as Behçet's disease to prevent delays in care. Early recognition and appropriate management of underlying conditions are essential for improving patient outcomes and quality of life. We present a case of a 41-year-old Scottish male who came in with complaints of recurrent oral ulcers and oral thrush. Initial treatment by an infectious disease specialist resolved the oral thrush but not the ulcers. Despite further treatment attempts for three years, including biopsy and antiviral therapy, ulcers persisted. Finally, referral to rheumatology led to comprehensive autoimmune testing, revealing positive HLA B51 and a diagnosis of Behçet's disease. Treatment with topical steroids and colchicine yielded significant improvement.

## Introduction

Behçet's disease (BD) is a chronic and relapsing vasculitis with potential multisystemic involvement. It is an idiopathic condition with a higher prevalence along the historic 'Silk Road,' spanning from Eastern Asia through the Middle East to the Mediterranean, in contrast to occurrences in Western nations, but has a universal distribution [1]. It is characterized by autoinflammatory vasculitis affecting arteries and veins of various sizes, with unique features such as venular involvement and the formation of pulmonary and arterial aneurysms. The histopathological characteristics include vasculitis and thrombosis. Unlike other types of vasculitis, BD does not display necrotizing vasculitis or the formation of giant cells [2]. The hallmark feature of BD is oral ulcers, with oral aphthosis observed in over 95% of patients and often serving as the initial presentation [3]. Aphthous ulcers manifest as painful oral sores resembling canker sores, and typically, they heal within one to three weeks but tend to recur. The primary affected locations include the gingival, buccal, labial mucosa, and tongue. Additionally, they may manifest in the soft and hard palate, oropharynx, and tonsils [4]. Differential diagnoses include viral, fungal, and bacterial infections, nutritional deficiency, trauma, drug-induced causes, immunological etiologies, and fever syndromes [5].

Oral ulcers are uncomfortable for patients and cause problems with chewing and eating food. This in turn leads to food aversion, depression, weight loss, and multiple other unpleasant conditions. Thus, delays in diagnosis can lead to significant morbidity and mental distress for the patients. This case report describes a patient with recurrent aphthous ulcers who underwent a comprehensive diagnostic investigation, including assessments for infectious causes. Ultimately, the diagnosis of BD was established. Our case report aims to encourage clinicians to consider Behçet's disease in their differential diagnosis when dealing with recurrent oral ulcers refractory to usual treatments to prevent delay in care. This article was previously presented as an abstract poster at the 2024 Society of Hospital Medicine (SHM) Converge National Meeting on April 14, 2024.

## Case presentation

A 41-year-old Scottish male patient, with a past medical history significant for erectile dysfunction, prior Epstein-Barr virus (EBV) infection, hyperlipidemia, umbilical hernia, and urethral meatal stenosis was referred by his primary care physician (PCP) to rheumatology for oral thrush and recurrent oral ulcers for three years. The patient was previously seen by an infectious disease doctor who reported the resolution of oral thrush with clotrimazole troches. However, oral ulcers remained and were refractory to nystatin and magic mouthwash. A physical exam of the oral ulcers revealed reddish oval lesions with a white center and erythematous border. A biopsy of the oral ulcer by an oral surgeon was non-diagnostic and negative for the herpes simplex virus (HSV). The patient later developed a burning micturition and a purple hue at the urethral meatus, along with a genital/scrotal ulcer. He was presumed to have a urinary tract infection (UTI) and was treated with ciprofloxacin, which resolved the dysuria but not the genital lesions. The patient was then referred to our rheumatology clinic for further evaluation to rule out an autoimmune etiology. Antinuclear antibody (ANA), anti-ds DNA, antineutrophil cytoplasmic antibodies (ANCA) screen with myeloperoxidase (MPO) and proteinase 3 (PR3), Coombs test, cytomegalovirus (CMV), hepatitis panel, and sexually transmitted infections (STI) testing-HIV, Chlamydia polymerase chain reaction (PCR), and rapid plasma reagin (RPR) were all negative, but HLA B51 resulted in a positive result. The diagnosis of Behçet's disease was made. Treatment with topical steroids (as needed) and colchicine (for prophylaxis) was initiated, showing a remarkable response.

## Discussion

Recurrent oral ulcers, often referred to as aphthous stomatitis or canker sores, are a common clinical complaint characterized by the appearance of painful, recurrent lesions in the oral mucosa. These ulcers typically present as small, round, or oval sores with a yellow or white center and a red border, causing significant discomfort and impacting daily activities such as eating and speaking [5]. While the exact etiology of these ulcers remains elusive, they are believed to result from a complex interplay of genetic predisposition, immune system dysfunction, and environmental triggers such as stress, hormonal changes, and certain foods [4].

The differential diagnosis for recurrent oral ulcers is broad and includes conditions ranging from benign and idiopathic to systemic diseases. Standard differentials include infections (viral, bacterial, and fungal), autoimmune disorders (such as Behçet's disease, systemic lupus erythematosus, and pemphigus vulgaris), nutritional deficiencies (vitamin B12, folate, and iron), and gastrointestinal disorders like Crohn's disease and Celiac disease. In some cases, oral ulcers may also be induced by medications, including nonsteroidal anti-inflammatory drugs (NSAIDs), beta-blockers, and angiotensin-converting enzyme (ACE) inhibitors [6]. Cases, where oral ulcers persist despite usual treatments with topical steroids, analgesics, vitamin supplementation, and antimicrobial mouthwashes, have also been identified. This persistence suggests further investigation into underlying systemic diseases or other contributing factors. Behçet's disease, in particular, is an important differential diagnosis to consider when patients present with recurrent oral ulcers that do not respond to standard therapies [7].

Behçet's disease is a chronic, multisystemic vasculitis of unknown etiology, characterized by recurrent oral and genital ulcers, uveitis, and skin lesions. The oral ulcers associated with Behçet's disease are similar in appearance to idiopathic aphthous ulcers but tend to be more numerous, larger, and more painful. The diagnosis of Behçet’s disease is primarily clinical, as there are no specific laboratory tests that can definitively diagnose the condition [8]. The International Study Group for Behçet’s Disease has established criteria that include recurrent oral ulcers at least three times in twelve months as a major symptom, along with at least two of the following: genital ulcers, eye inflammation, skin lesions, or a positive pathergy test, which is a skin prick test that can cause a specific red bump to form in patients with Behçet’s [9]. Clinical features of Behçet’s disease are mentioned in Table 1. Per the International Criteria for Behçet’s disease scoring system (Table 2), patients with ≥4 points have been classified as having Behçet’s disease. Our patient had recurrent oral ulcers and genital ulcers, accounting for 4 points, and has been diagnosed with Behçet’s disease. These criteria help clinicians identify the disease in patients presenting with symptoms that could be attributed to several other conditions [9]. The diagnosis can be particularly challenging when oral ulcers precede other symptoms by years, requiring clinicians to maintain a high index of suspicion and a thorough patient history.

**Table 1:** Clinical manifestations of Behçet's disease [10]

Systems	Signs and Symptoms
Oral	Recurrent aphthous ulcers
Urogenital	Genital ulcers
Ocular	Anterior/posterior uveitis, retinal vasculitis
Skin	Erythema nodosum, pseudofolliculitis, acneiform nodules, papulopustular lesions
Neurology	Cerebral venous thrombosis, headache, disorientation, less commonly stroke
Musculoskeletal	Arthralgia, asymmetric arthritis
Vascular	Arterial thrombosis, aneurysm, venous thrombosis
Gastrointestinal	Abdominal pain, diarrhea, bleeding
Non-specific	Fever, fatigue, malaise

**Table 2:** The International Criteria for Behçet disease scoring system Point score system: scoring ≥ 4 indicates Behçet’s diagnosis [9]

Signs and Symptoms	Score
Ocular lesions	2
Genital ulcers	2
Oral aphthous ulcers	2
Skin lesions	1
Neurologic manifestations	1
Vascular manifestations	1
Positive pathergy test	1

The management of recurrent oral ulcers, particularly in the context of Behçet's disease, requires a multifaceted approach that addresses both the symptomatic relief of the ulcers and the underlying systemic inflammation characteristic of Behçet's [9]. For the general treatment of recurrent oral ulcers, options include topical therapies such as corticosteroid creams, gels, or mouthwashes that can reduce inflammation and pain. Topical analgesics like benzocaine or lidocaine can relieve temporary pain, and mouthwashes with ingredients like salt water or baking soda may help soothe the affected areas.

Treatment is more specialized in Behçet's disease, where oral ulcers are a hallmark symptom. Topical corticosteroids are often the first line of defense for managing mouth ulcers [11]. For more severe cases, systemic medications may be necessary. Apremilast (Otezla), which is FDA-approved for treating oral ulcers in Behçet's, can be effective [12]. Additionally, moderate doses of systemic corticosteroids like oral prednisone are frequently required during disease exacerbations. Some patients may need chronic, low-dose prednisone or conventional immunosuppressives such as azathioprine to keep the disease under control [11]. Other systemic interventions for managing oral ulcers in Behçet's disease include medications like acyclovir, thalidomide, and colchicine and biologic agents like etanercept and interferon-alpha. These treatments aim to reduce the frequency and severity of ulcer outbreaks and manage the systemic inflammation that drives Behçet's disease [11]. Maintaining good oral hygiene is also crucial for patients with Behçet's disease to prevent the exacerbation of ulcers and secondary infections. Patients are advised to brush and floss regularly, even when experiencing pain from oral ulcers. In addition, the use of toothpaste formulated for sensitive mouths or without sodium lauryl sulfate (SLS), which can irritate ulcers [13], is recommended.

## Conclusions

In summary, recurrent oral ulcers are a common yet complex condition with a wide range of potential etiologies. When ulcers persist despite conventional treatment, it is crucial to consider systemic diseases such as Behçet's disease. Early recognition and appropriate management of underlying conditions are essential for improving patient outcomes and quality of life. The treatment of recurrent oral ulcers, especially in the context of Behçet's disease, involves a combination of topical and systemic therapies tailored to the severity of the symptoms and the overall clinical picture of the patient. The metaphor of "horses" and "zebras" in medicine reminds us that while common conditions are more likely (horses), clinicians must also be vigilant for less common but significant diseases (zebras) like Behçet's disease, which can present with common symptoms like oral ulcers.
